# Bumble Bee Foraged Pollen Analyses in Spring Time in Southern Estonia Shows Abundant Food Sources

**DOI:** 10.3390/insects12100922

**Published:** 2021-10-09

**Authors:** Anna Bontšutšnaja, Reet Karise, Marika Mänd, Guy Smagghe

**Affiliations:** 1 Institute of Agricultural and Environmental Sciences, Estonian University of Life Sciences, 51006 Tartu, Estonia; marika.mand@emu.ee; 2 Laboratory of Agrozoology, Department of Plants and Crops, Ghent University, 9000 Ghent, Belgium; guy.smagghe@ugent.be

**Keywords:** bumble bees, food source richness, pollen determination with microscopy and DNA metabarcoding

## Abstract

**Simple Summary:**

Pollinators make a strong contribution to ecosystem stability. However, nowadays, they also need protection and sustainable habitat to live and develop. Not all regions can provide suitable habitats due to agricultural intensification, urbanization, climate changes and corresponding impacts. Our study was conducted in the late spring in south Estonia where arable lands were surrounded by forest patches and rural areas. For better performance, we used both light microscopy and DNA metabarcoding methods for pollen identification. We found that bumble bees foraged on the diverse food sources showing preferences for several main plant families. Additionally, in our case, land-use types did not show important effects on bumble bee food choices and foraging decisions. Various landscape features can provide diverse food sources at the early development stages and support nest longevity. Here, we can say that a better understanding of pollinators’ food preferences can help in the application of more suitable measures for their conservation.

**Abstract:**

Agricultural landscapes usually provide higher quantities of single-source food, which are noticeably lacking in diversity and might thus have low nutrient value for bumble bee colony development. Here, in this study, we analysed the pollen foraging preferences over a large territory of a heterogeneous agricultural landscape: southern Estonia. We aimed to assess the botanical diversity of bumble bee food plants in the spring time there. We looked for preferences for some food plants or signs of food shortage that could be associated with any particular landscape features. For this purpose, we took *Bombus terrestris* commercial hives to the landscape, performed microscopy analyses and improved the results with the innovative DNA metabarcoding technique to determine the botanical origin of bumble bee-collected pollen. We found high variability of forage plants with no strong relationship with any particular landscape features. Based on the low number of plant species in single flights, we deduce that the availability of main forage plants is sufficient indicating rich forage availabilities. Despite specific limitations, we saw strong correlations between microscopy and DNA metabarcoding data usable for quantification analyses. As a conclusion, we saw that the spring-time vegetation in southern Estonia can support bumble bee colony development regardless of the detailed landscape structure. The absence of clearly dominating food preference by the tested generalist bumble bee species *B. terrestris* makes us suggest that other bumble bee species, at least food generalists, should also find plenty of forage in their early development phase.

## 1. Introduction

Healthy pollinator assemblages in the vital beekeeping sector is recognized as critical to sustainable ecosystems [1]. The new realities imposed by climate changes necessitate the present-day understanding of the actual status of available resources among our landscapes to create or preserve areas really needed by pollinators. Smart land use is part of climate policy [2].

The importance of diet heterogeneity is variable by pollinator species, but improves their development and sustainability. The high-quality diet not only supports nutrition but also positively affects the bee immune system suppressing the negative impact of toxins and pesticides present in our landscapes [3]. Moreover, food source variability allows pollinators to make quality and quantity decisions according to forage preferences and behaviour [4,5,6]. Generalist pollinators can show food plant constancy by foraging on a limited number of certain plant families, which provide highly valuable nutrient content [7,8]. These highly attractive families often support several bee species. Knowing the food plant availabilities in specific landscapes would be an important measure for wild pollinator conservation.

Agricultural landscapes usually provide higher quantities of single-source food, which are noticeably lacking in diversity [9]. Seminatural areas thereafter might support the bees with food of higher value. Smart et al. [10] demonstrated that the lipid content of honey bee (*Apis mellifera* L.) abdomens was positively correlated with the proportion of seminatural landscapes. While seminatural and even natural landscapes are tightly related to agriculture and pesticide residues are known to affect organisms even far away from fields, high-value food can relieve the negative impacts of the chemicals on bees [11]. 

Several interventions have been developed to sustain pollinator-friendly agro-ecosystems [12]. The unmanaged grasslands often turn to hay without interference; the field boundaries need attention to prevent the growth of invasive weeds or hay. Marja et al. [13] described that even if the intervention does not lead to major changes, these help to preserve the situation. The crops themselves support bee species differently, while the foraging strategies and dependence on alternative plant species differ much from one bee species to another. Raimets et al. [14] even demonstrated that within the same landscape, honey bees were less attracted to spring compared to summer flowering oilseed rape. This allows suggesting that the plant species availability was better in spring time and the pressure to fly long distances was lower. Bumble bees, however, fly notably shorter distances [15,16] which limits the access to rich flower resources and increases the environmental pressure on colony development.

One way to assess the forage plant availabilities is to use bee-collected pollen. In the past, plant species identification has been performed only by light microscopy, which is very laborious, and usually, the identification stays at the family level [17]. Nowadays, more accurate methods are available. Consequently, the traditional techniques could be supplemented by innovative DNA metabarcoding techniques [18,19,20]. Plant species taxonomic identification has been achieved by using, for instance, the second internal transcribed spacer (ITS2), a widely used marker, and constantly updated databases of sequences. ITS2 should discriminate not only plant families but also differentiate between plant species allowing going deeper with pollen analyses. However, there is still no correct calibration of the numbers of sequence reads from DNA metabarcoding to actual proportions of pollen grains in the sample. Some studies state that the relative amounts of reads of any particular taxa are comparable to their actual proportions [21,22], but this might be organism-group or taxa-specific [23,24]. Thus, until now, the DNA metabarcoding for processing mixed samples has been used, rather, to obtain a broader overview of the pollen taxonomic origin but exclud quantity assessments [18,23]. Shortcomings also occur with microscopy technique as this does not determine every single pollen grain in a sample, which is why this technique might miss too many plant species limiting the accuracy of proportional estimations, too. Reliable determination of plant species proportions in bee-collected pollen samples is an approach that needs further development.

Here, in this study, we aimed to assess the botanical diversity of bumble bee food plants in the spring time in heterogeneous landscapes of southern Estonia. We used bumble bee hives to assess the landscape for pollinator support as described by [25]. We wanted to see whether there is a preference for some food plants or signs of food shortage that could be associated with any particular landscape features. In addition, we raised the question about the comparability of microscopy and DNA metabarcoding techniques in the determination of the botanical origin of bumble bee-collected pollen.

## 2. Materials and Methods

### 2.1. Study Environment 

**Figure 1:**
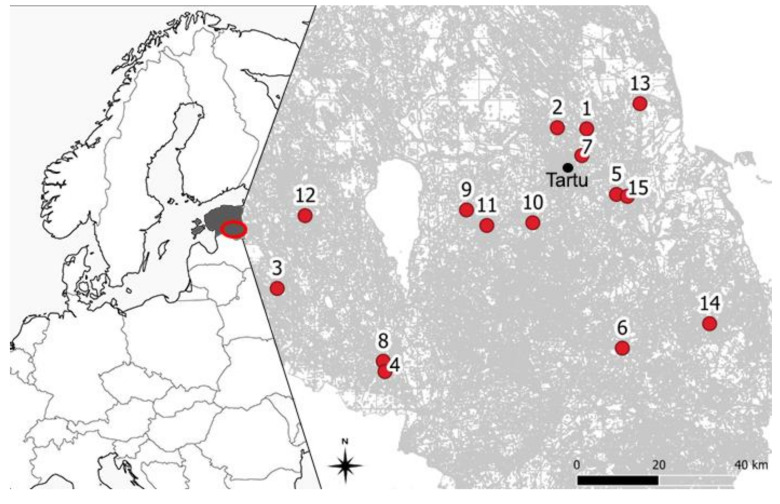
A schematic overview of the location of the study area on the European map (**left**) with detailed southern Estonian region (**right**) zoomed out. The 15 study locations around Tartu city are indicated with red circles and numbers.

We selected 15 sites for our study in 2017 to understand the availability of forage resources in southern Estonia (Tartumaa, Viljandimaa, Põlvamaa and Võrumaa counties) (Figure 1). Sites were chosen according to variability, logistical limitations and local owners’ agreements. The region is characterised by heterogeneous landscapes (examples are presented in Supplementary materials, Figure S1a–c), where rather extensive agriculture is practiced. The landscape is slightly hilly, with lots of lakes, rivers and forest patches. Due to high patchiness, the local weather conditions create suitable microclimates for growing fruits and berries. The human inhabitation is rather low, 10–20 residents per km^2^ [26], but still evenly distributed. The density of households is scattered [27], mostly single-family houses. The houses are surrounded by yards, where fruit trees, flowerbeds and berries are common. Although a large share of Estonian territory is covered with forests, there are plenty of forest patches, clear-cuts, and brush. In spring time these landscapes usually are with plenty of flowers different trees and berries, ornamental plants in yards, small flowers on underwood and early season flowering plants on field edges and meadows. 

### 2.2. Hive Locations

Thirty bumble beehives were located in various environments with one common feature, strawberry field, next to the hives. We selected 15 sites; in each, we located two hives next to each other with about a 1 m gap between them. We located them on the strawberry field borders so that at least 50% of the foraging territory was a non-crop area. Strawberry was chosen to guarantee at least one food source for our experimental colonies. While strawberry is not the preferred food source for *Bombus terrestris* [28,29], we expected bumble bees to forage on more suitable plants with the same flowering period. Thus, the high strawberry pollen proportion might have reflected an absence of more suitable food sources.

We searched for contrasting landscapes in 1000 m radiuses around beehives based on a share of different land-use types. The 1000 m radius was selected, because it is commonly the maximum bumble bee foraging range [15,16], although sometimes few marked bumble bees have been seen also at a distance of 1500 m [30]. The site selection excluded foraging area overlapping. Distances between study sides varied between 3.7 km and 104 km (Figure 1).

**Figure 2:**
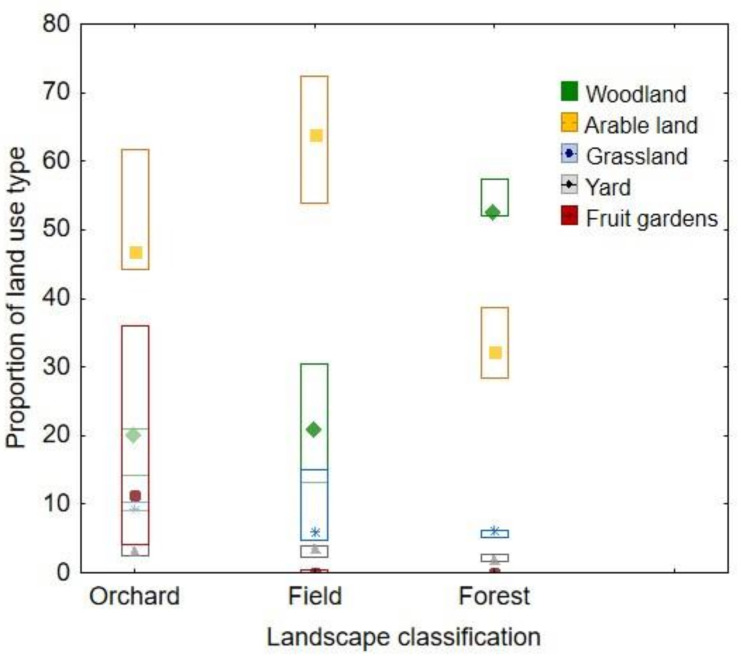
The share of land-use types (woodland, arable land, grassland, yards and fruit gardens) across the three land-use categories (Orchard, Field and Forest) within a radius of 1000 m around hives.

We used maps of Estonian Land Board and QGIS software (QGIS Development Team, 2018. QGIS Geographic Information System. Open Source Geospatial Foundation Project. http://qgis.osgeo.org, accessed on 2 August 2021) to calculate the proportions of land use characteristics around the bumble bee nests to understand which land-use types better explain the pollen choice of bumble bees. The characteristics used were: shares of woodland, arable land, grassland, yards and fruit gardens. While no clear patterns occurred when using these land-use parameters in statistical analyses, we additionally divided the land use parameters into the categories based on the three most important ones and named them as Forest (5 sites), Field (7 sites) and Orchards (3 sites) (Figure 2, illustrative material provided in supplementary materials). When a site had more than 50% of the area covered by woodland, it was classified to Forest category; when more than 50% was covered by arable lands, it was classified to Field category. The Orchard category was very variable while fruit gardens are mostly located in agricultural landscapes, which explains the large share of “arable land”; the share of “woodland” stayed around 20% there. Preliminary statistical analysis showed that land-use types “grasslands” and “yards” did not differ between the categories (grasslands: *p* = 0.91, yards: *p* = 0.22).

### 2.3. Bumble Bees

The bumble bee *Bombus terrestris* L. is a common short-tongued bumble bee with a broad food plant spectrum. *B. terrestris* is a species that is related to open landscapes and its abundance is negatively correlated with the share of woodlands [31]. *B. terrestris* is considered a spatial generalist because of its large foraging distances [32,33]. They might prefer open landscapes, but forest patches are not barriers [34]. This species has relatively large colonies, therefore they need rich foraging areas to cover the needs of the developing brood.

Commercially reared bumble bee *B. terrestris* hives were obtained from Biobest, Westerlo, Belgium. The hives were Standard queenright colonies with about 80 workers at the beginning of the experiment. The hives were equipped with sugar solution (Biogluc bottle, from the producer) and polystyrene outer coating (Bee-Coat, from the producer) to keep the colonies warm even in cool nights which may occur in this time of year. 

Before taking the colonies to the field, they had no previous foraging experiences in these landscapes nor with these local plant availabilities. If the bumble bees had developed some food preference based on the food the hives were provided during the early development of the colonies [35], we suppose all colonies had similar experiences. The hive entrances were opened two days before sampling of pollen, to allow foragers to learn the foraging territory and food plants available. 

All the colonies used were in their developing phase and while all the food provided by the producer was eliminated, we expected them to forage actively. All hives were weighed before and after the experiment and the weight increase was obvious 119.6 ± 17.1 g. All hives were in the middle of the development stage (brood cells were present, no gynes or males detected).

### 2.4. Collection of Pollen

Pollen sampling was timed to the mid flowering of strawberry fields to guarantee the highest number of strawberry flowers available. Pollen was collected twice from each hive and location with an in-between period of 5–7 days, covering the whole strawberry flowering period. Moreover, at the same time orchards are in their late flowering state, winter oilseed rape is in the middle or final stage of flowering depending on the cultivar. Additionally, natural plants provide high flower density. The foraging activity of bumble bees from different hives was variable, therefore the number of pollen samples varied, being 4–10 pollen loads from individual bumble bees. Each sampling lasted roughly one hour per site between 9:00 to 18:00. Each hive was monitored once in the morning and once in the afternoon and thus we suppose the time of day did not affect the outcome. Both pollen loads were collected from the homing foragers. The hive entrances were closed with plastic jars and the bee was cached into it. Each jar was thoroughly cleaned after every usage to avoid cross-contamination of the samples. Bumblebees were released after pollen loads were taken. Individual forager pollen loads were packed into paper bags and labelled with the date, time, hive number and study site. In total, 427 samples were collected during the study period with a mean of 8.9 ± 0.13 individuals from each hive each date. Bags were air-dried at room temperature, then the pollen loads consisting of two pollen pellets were weighed and separated into two Eppendorf tubes one for light microscopy and the other for DNA metabarcoding.

### 2.5. The Plant Species Determination of Pollen

#### 2.5.1. Light Microscopy

For light microscopy, pollen grain structure needs to be recognisable. We followed the protocol described by [36] to purify the pollen pellets from excessive materials. For that 1 mL of 99.6% acetic acid was added for 48 h to separate the pollen kit and to clarify pollen grain external structure. After that, the solution was homogenized by a glass stirring rod and centrifuged for 5 min at 13,400 rpm. For the next step, acetic acid was discarded and replaced by 1 mL of the demineralised water. Afterwards, samples are centrifuged for 5 min at 13,400 rpm, demineralised water was changed and samples were stored at room temperature. Then, the sample was transferred to a slide by the glass stirring rod and covered by the cover glass without adding any dye. From each sample, 200 pollen grains were determined at 400× *g* magnification (Olympus CX 31 RBSF) to genus level, and where possible to species level, using 1000× *g* magnification (Nikon H550L) in cases, where the structure was not seen or ambiguous. As references were used flowering plants (about 70 plant species) pollen collected from the study sites during the field-work period and prepared by the same protocol. An example of the strawberry pollen grain seen through light microscopy is presented in Supplementary materials Figure S2. Reference publications were also used [36,37].

#### 2.5.2. DNA Metabarcoding

For the first step of DNA metabarcoding, dried pollen was pooled from the same hive and day to obtain a larger sample size. After that mixed pollen was homogenised by mortar. In total, 54 samples were used for DNA extraction. An amount of 50 mg of powdered pollen was placed into a 2 mL Eppendorf tube, adding 800 µL of RTL buffer and 8µl of beta-mercaptoethanol, after 5 s vortex, 0.3 g of silica beads were added. Samples were lysed (2 min at 30 Hz) and homogenised (2 min 20 Hz) using the TissueLyser II (Qiagen, Venlo, the Netherlands). Subsequently, samples were centrifuged for 2 min at 20,000× *g* and 200 µl supernatant was used for DNA extraction. DNA was extracted using the Invisorb Spin Tissue Mini Kit (Stratec molecular GMBH, Berlin, Germany) according to the manufacturer’s protocol No. 5. In between work, samples were stored at −20 °C. After DNA isolation, the ITS2 region was amplified by PCR (polymerase chain reaction) using dual-barcoding as described by [20]. Each sample was amplified in triplicate. Samples were initially denatured at 95 °C for 4 min, then amplified within 36 cycles at 95 °C for the 40 s, 49 °C for 40 s and 72 °C for 40s with a 5min final extension (72 °C). After PCR amplification, replicates were pooled and quantified using the Quan-IT™ PicoGreen™ dsDNA kit (Thermo Fisher Scientific, MA, USA). The quantified samples were normalized and subsequently purified and concentrated using E.Z.N.A.^®^ Cycle Pure Kit (Omega Bio-Tek, Norcross, GA, USA)and AmiconUltra-0.5 columns (Millipore Cooperation, Billercia, MA, USA), respectively. Samples were sent for sequencing using Illumina Miseq PE250 (NXTGNT, Ghent University, Ghent, Belgium).

Raw reads were combined and the quality was checked using MOTHUR 1.34.4 [38]. Combined reads were filtered on zero ambiguity and amplicon length between 100 bp and 360 bp. Unique sequences were blasted against a custom database that contained the ITS2 region of plants (compiled from [39]). Clustering was performed on 97% identity. The most abundant sequence of each cluster was used as a representative sequence which was identified to the genus level using the Blast function in NCBI. We looked at the results to filter out plants not occurring in Estonia.

### 2.6. Statistical Analyses

We assessed five land-use parameters in the 400 m and 1000 m radius of the hives: woodland, arable land, grassland, yards and fruit gardens. Exact proportions of different land-use types were calculated using the QGIS (version 3.4.2), Microsoft Excel 2016 (Microsoft, Washington, DC, USA) software and Estonian Basic Map 1:10,000 (Estonian Land Board 2018).

One way ANOVA was used in analyses of variability between different sites or landscape categories at levels of plant families and genera, while Wald test was used in analyses at the plant species level. Kruskal–Wallis median test was used to estimate differences in strawberry pollen abundance between landscape categories and confirm land use parameter differences between selected landscape categories, *Fragaria* reads or Strawberry pollen counts in landscape categories. Pearson’s correlation coefficient r was used to measure the strength of relationships between the proportions of strawberry pollen and diversity of plant families foraged, between proportions of strawberry pollen and *Fragaria* reads; between proportions of plant families and land use parameters (see Supplementary materials Table S1 for all the r-value color codes of all the individual correlations). All the analyses were made using statistical software Statistics version 13 (© 2021 StatSoft Europe, Hamburg, Germany).

## 3. Results

### 3.1. Taxonomic Variability

**Figure 3:**
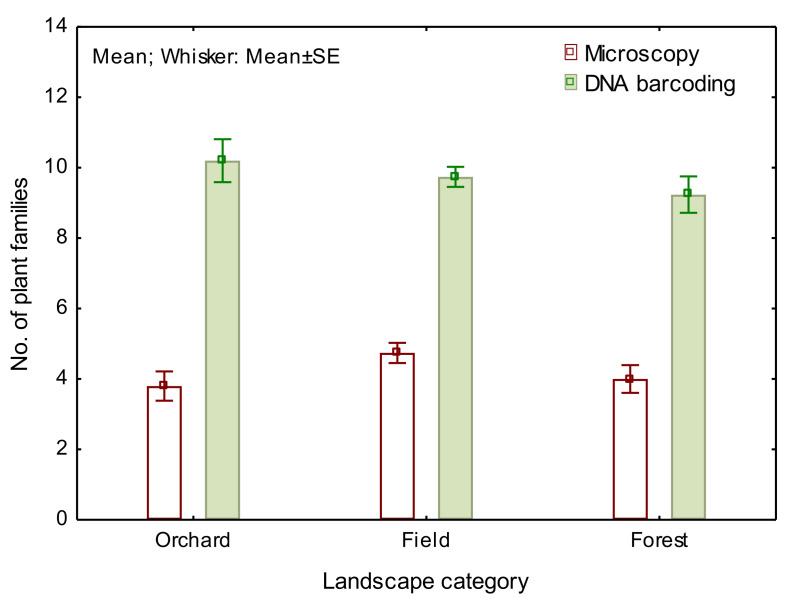
A number of plant families were determined with microscopy and DNA metabarcoding in bumble bee pollen forage from different landscape categories.

We compared the study sites and thereafter three landscape categories at different plant taxonomic levels: family, genera, and species. The microscopy technique helped us to define on average 4.3 ± 0.2 SE plant families from each hive and date, a result, which is low compared to the 9.7 ± 0.2 SE determined with DNA metabarcoding. The innovative technology allows us to seek deeper into the dataset. Using DNA data, we defined pollen from 18.9 ± 0.4 SE plant genera. There was no variation between sites nor landscape categories (Figure 3, Table 1). We saw that despite being also geographically distinct, the numbers of plant families and genera foraged in each site or landscape category did not differ (except at the site level according to the DNA barcoding results).

The data pooled over hive, site and date showed that although the maximum proportion of strawberry pollen was 64.8%, the median was as low as 5.6 % ± 20.5 SD. There was no correlation between the proportion of strawberry (determined by microscopy) foraged and diversity of plant families foraged (microscopy: r = 0.06, *p* = 0.68, r^2^ = 0.003; DNA barcoding: r = 0.02; *p* = 0.91, r^2^ < 0.001) and no difference between landscape categories (KW-H(2; 54) = 2.12, *p* = 0.35). There were significant correlations between some land-use types and strawberry foraged: the abundance of yards tended to decrease and the abundance of fruit gardens increased strawberry pollen forage (Supplementary materials Table S1).

**Figure 4:**
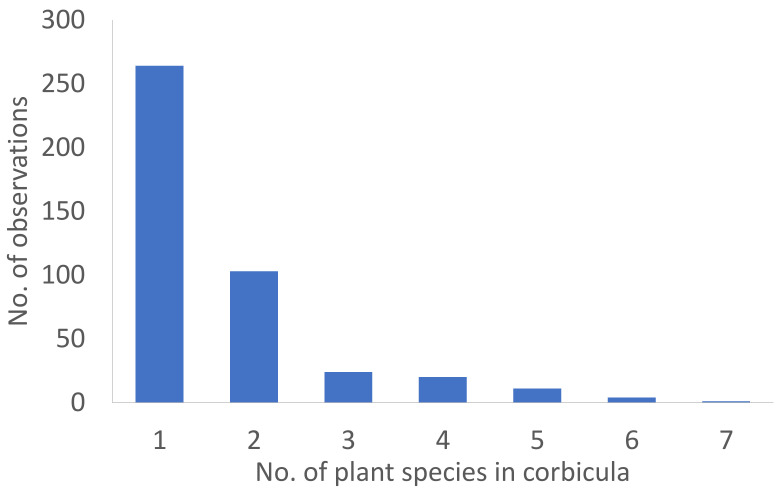
The number of bumble bee corbicular pollen loads containing a different number of plant species.

The median number of plant species per single foraging flight (analysed by microscopy technique) was 1.65 ± 1.09 SD (Figure 4) with no significant difference neither at site nor landscape category level (Table 1). Although the strawberry was the closest pollen source available in each site, it was not preferred by bumble bees. Considering samples, where one species formed more than 70% of all pollens determined, it was strawberry only in 15% of samples, while being Rosaceae in 24% and Fabaceae in 35% of samples. Both main plant families were accompanied by several plant Families. However, the Rosaceae was accompanied by Fabaceae in 54% of cases, but Fabaceae was accompanied with Rosaceae only in 23% of cases.

### 3.2. Proportional Variability of Pollens between Landscape Categories (Plant Family Level)

**Figure 5:**
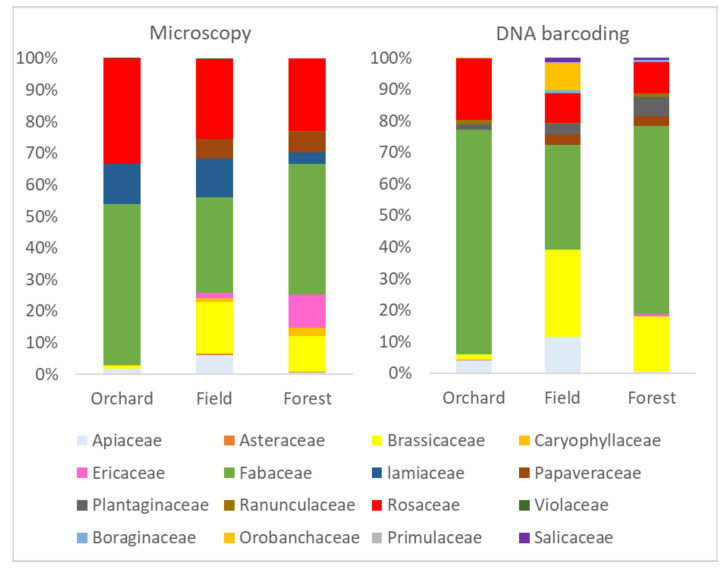
The percentage of bumble bee corbicular pollen loads containing the different number of plant species.

Because both of the techniques have limitations in their plant species determination ability, we made the quantifying analysis at the level of plant families. We saw three major plant families represented in pollens foraged by bumble bees. These were Fabaceae, Rosaceae and Brassicaceae. Although there were some differences in quantification reliability between the two pollen identification techniques (detailed analyses are presented in Supplementary materials Figure S3), we saw many variations between sites, but much less between landscape categories (Statistical details presented in Supplementary materials Table S2). Both techniques indicated the most abundant plant family in bumble bee forage being Fabaceae, which was prevailing in Orchards and least common in Field. Brassicaceae was prevailing in landscape category Field, less, but still abundant in category Forest, but almost absent in Orchards, surprisingly (Figure 5). Although the detection rate of Rosaceae pollens was significantly lower with DNA metabarcoding. There were no differences between landscape categories. The DNA metabarcoding detected four less represented families in addition to those identified with microscopy. The Lamiaceae pollen, which comprised even more than 40% of total pollen counts in some hives and dates by microscopy, was almost absent based on DNA metabarcoding. The same with Ericaceae pollen, which was detected in extremely low levels with DNA metabarcoding, although the microscopy technique showed high prevalence in one forested site.

### 3.3. Correlations of Pollen Proportions and Share of Land Use Parameters (Plant Family Level)

Correlation analysis with proportions of pollens collected and share of land-use types (Supplementary materials, Table S1) in each site at radiuses of 400 m and 1000 m showed few significant results: positive correlation occurred between forests and Ericaceae, grasslands and Brassicaceae, yards and Fabaceae at 400 m radius. Lengthening the radius added positive correlations between arable land and Apiaceae and also Lamiaceae. Negative correlations occurred between orchards and Brassicaceae, arable land and Ericaceae, yards and Rosaceae at 400 m radius. At 1000 m radius, also between yards and Ericaceae, forests and Lamiaceae, grasslands and Rosaceae. Based on microscopy data, the number of families and share of grasslands within 400 m radius showed a significant positive correlation, however, this significance got lost when analysed with DNA metabarcoding or when the distance estimation was lengthened. The pollens of less preferred plant families Orobanchaceae and Primulaceae showed also a significant positive correlation with grasslands and yards in a shorter distance.

### 3.4. Reliability of DNA Metabarcoding Quantification

We saw good correlations between microscopy and metabarcoding data (look at Supplementary materials Figure S2). Some plant families tended to show positive correlations between the results from microscopy pollen grain counting and relative proportions of reads from DNA metabarcoding. Best overlaps between these two datasets were revealed with these families, which are the most probable forage plants for bumble bees and were also most commonly detected in this study. These families are Brassicaceae (b = 1.02, *r* = 0.48), Fabaceae (b = 0.99, *r* = 0.70) and Rosaceae (b = 0.56, *r* = 0.65). Good correlations were detected also in Apiaceae (b = 1.13, *r* = 0.50) and Papaveraceae (b = 0.58, *r* = 0.87), but these were of minor importance to bumble bees. In comparison with the microscopy technique, the DNA metabarcoding showed a proportional detection rate only for Brassicaceae and Fabaceae, while overestimating Apiaceae, Plantaginaceae and Ranunculaceae pollens. Rosaceae, Papaveraceae. Asteraceae, Caryophyllaceae, Ericaceae and Lamiaceae were underestimated.

## 4. Discussion

Based on the botanical diversity of bumble bee foraged pollens we confirmed that the heterogeneous agricultural region of southern Estonia supports bumble bees well in spring time. We saw the highly variable food availability over the large study territory and no particular preference for any single plant family. At the same time, the data of individual forage flights suggest sufficient availability of the bumble bees’ most preferred plant species.

Many bumble bee species are generalists and may forage on almost any entomophilous plant species available. The quality and availability of spring time forage might be limiting factors for the persistence of rich bumble bee fauna [40]. The *Bombus terrestris* forms large colonies naturally and the foragers are evolved to perform flights, starting with hundreds of meters from the nest, allowing to search a large territory around [30]. We used fully developed *B. terrestris* colonies to achieve the proper sampling of flower resources at the time. Natural bumble bee colonies of any species are at their early development phase and are not so numerous to outcompete our test bumble bees. 

Plant taxonomic composition and proportional content of pollens collected by bumble bees can give an overview of the forage plant richness of particular environments. Although generalist bumble bees can forage on a large variety of plant species. Each individual bee learns and remembers few rewarding flower types to diminish the energetic costs of foraging [41,42]. Here, a number of workers, as a feature of the developmental stage of the colony, can affect at some point not only food preferences [43] but also foraging intensity. Parmentier et al. [25] show the relatedness of bumble bee preferred pollens to the availability of these plants in bee foraging areas and suggested this phenomenon to be ever-changing and dependent on the season. In case of abundant and diverse forage availability, the flower constancy develops to increase the foraging effort [44] and the poorer the resources, the stronger the pressure to forage on a higher number of plant taxa. The diversity of plant species in bumble bee pollen pellets may indicate the quality of anthropogenic landscapes to bumble bees [25]. Hence, our results here indicate sufficient food source variability over all the study sites. While Fabaceae and Rosaceae were the most preferred plant families, as common, the accompanying plants in bumble bee pollen pellets were different for the two main plant families. Fabaceae were more often accompanied by other plants than Rosaceae, while in more than half of cases the Rosaceae were accompanied with Fabaceae. This might come because of differences in nectar and pollen nutritional value [45]. As Fabaceae plants live in symbiosis with nitrogen-fixing bacteria, their nectar and pollen can be with higher nitrogen content compared to other plant species through a better (self)-fertilisation rate [46]. Flower resources, like pollen and nectar, can benefit from specific fertilisation also in other plant species [47]. Fabaceae plants are often seen to be very attractive to many bumble bee species [48,49,50], including *B. terrestris* which was used in this study. Pollen nutritional value of Rosaceae plants has been estimated twice as low as that of Fabaceae when estimated by the protein:lipid ratio [51]. Despite the lower nutritional value, the availability of Rosaceae was higher (the strawberry!). Moreover, in Estonia, many spring-flowering fruit trees and berries, including strawberries, belong to the Rosaceae family.

The spring time floral richness offers plenty of flowering plants and as bumble bees prefer to switch between plants, they do not rely on massive crops. Kallioniemi et al. [52] showed that the flowering crops have a negative effect on bumble bees in the spring time but a positive effect in summer. Our results confirm that bumble bees were not interested in crops at this particular time of year. Among Brassicaceae plants, the oilseed rape and turnip rape are commonly grown, but our data indicated that rather the weeds were prevailing in bumble bee forage. Wild species of Brassicaceae prefer open landscapes and are flowering on wastelands, grasslands and inside cereal fields, where these may also form massive flower resources. In our case, while in the study region, also beekeeping is common, the honey bees may have outcompeted bumble bees from larger patches of Brassicaceae, either weeds or crops. Raimets et al. [14] sampled honey bee hives during the flowering of winter oilseed rape, which falls pretty much into the same time frame as our study, and saw that Brassicaceae formed more than a half of foraged pollens.

Lamiaceae are often highly attractive for several species of bumble bees in spring time. In our study, we saw that in some study sites bumble bees foraged on it, but the proportion stayed low. It might come from the unsuitable weather conditions the study year was with rather cool and moist weather, which suppresses the nectar production of this plant [53].

Many studies point to the importance of different land-use types. Flower-rich field margins are shown to be a great food source for bumble bees [54], but this was not confirmed in our study. We saw that proportion of arable land was positively related only with Apiaceae, Lamiaceae and Brassicaceae. The last one belonged to the three most commonly foraged plant families, but was clearly less attractive compared to Fabaceae and Rosaceae. Lye et al. [55] argued that flower-rich grasslands support bumble bee colony initialisation and early development, due to suitability for nesting and foraging and. In our study region, however, the proportion of grasslands supported only forage on Brassicaceae and some minor importance families. Mola et al. [56] demonstrated the importance of herbal plants flowering in forest underwoods at the period of young queens establishing their nests. Osborne et al. [57] found the yards being valuable nesting sites, which simultaneously provide variable forage resources. We saw the relationship of yards and Fabaceae and some other less attractive plant families. The yards might be of higher importance when natural forage is scarce. Surprisingly, in spring time, fruit and berry gardens did not support the forage of Rosaceae pollens. Instead, the Fabaceae was prevailing there like everywhere else. We are not opposed to all aforementioned studies, but we show that every study region, plant communities and particular time windows design their datasets and outcomes. Different habitat types are important at the different time points and bumble bee development stages. Indeed, our study should be continued to estimate the value of this environment throughout the season.

Further improvements are still needed for technical gaps in the quantification of bee-collected pollen samples. In this study, we found at least partial overlap in the results. Although light microscopy and DNA metabarcoding are powerful methods for insect collected pollen identification applying to biological, ecological and even agricultural studies [58,59,60], both have their limitations. Light microscopy provides more accurate quantitative results, but it takes a lot of time to confirm identification and needs to use different supporting materials and techniques (such as reference samples and an electron microscope). While it is not realistic to identify every single pollen grain, less represented species could be non-detected. DNA metabarcoding produces qualitative information about all plant species found in the pollen sample. Many samples could be processed with a little effort, but thereafter a wide range of the information needs to be analysed to exclude DNA reads, pointing on plants not available in the region or not flowering at the research period. Bell et al. [23] also showed the misinterpretation of related species because of similar nucleotide sequences in the target site of the genome. Based on our results, the DNA metabarcoding did not separate crops like strawberry nor oilseed rape, which is feasible with microscopy (see strawberry pollen illustration in Supplementary materials). Instead, DNA metabarcoding suggested the wild relatives of these plants. As an improvement, the DNA metabarcoding allows to use of plant genera at a taxonomic level in the analyses, this would be hard to achieve with microscopy by large datasets. The DNA metabarcoding is not well calibrated yet to allow a quantitative approach in studies [18,23]. Just like Bell et al. [23] described, our data also show strong over and underestimation of some particular plant species. Bell et al. [23] reviewed that the difficulties in quantification in DNA metabarcoding studies might occur due to a number of copies of the DNA regions, preservation, DNA isolation and amplification biases. This might be affected by several factors, and even by chloroplast inheritance modes maternal, paternal and biparental between different plants. Still, we claim that in the most important bumble bee forage plants, the proportional quantification of the reads produces a fairly good result. Bänsch et al. [61] also used the DNA metabarcoding with reads from ITS2 region sequences to quantify the bumble bee-collected pollen but reminded that the interpretation must take the specific limitations of the outcome into account.

## 5. Conclusions

The availability of nesting sites and food plants can be scarce in modern landscapes. Urbanization gathers people to cities and suburban areas, and thus, populated rural areas are declining. The landscape in southern Estonia represents rural municipalities where the agricultural activity is present but with lower intensity and scattered small settlements are alternated with forest patches, meadows and fields. This region is also rich in traditional villages with privately owned small vegetable and fruit gardens. The disappearance of dairy cattle and unification of smaller agricultural enterprises to larger ones are occurring here like elsewhere alongside modernisation in both agriculture and forestry. These processes usually bring the homogenisation of landscapes and the decrease in the availability of food plants of pollinators. 

In order to achieve stable pollinator fauna, we need to understand the parameters supporting them. The needs of bees are described, but the ever-changing conditions are to be overlooked. Until recent times, environmental protection has been focused mainly on preserving certain best suitable areas, often represented by small habitat islands with or without connections between them. Instead of creating a dense network of small various habitats, we should look at larger regions entirely supporting pollinators. As the study of Mola et al. [55] pointed, some habitat types have gained most of the attention and some others have remained disregarded. Based on the Estonian bumble bee monitoring dataset, it is shown that agri-environmental schemes determining the frequency of legume crops in crop rotations and size of field margins [13] most probably support general bumble bee fauna; however, perhaps not for all species. Our study focuses on a small time frame in spring which is most important to support bumble bee colony initialization. If the environment in southern Estonia also supported late-season forage, this region could serve as the bumble bee’s best habitat and national rural development plans should consider this.

## Figures and Tables

**Table 1:** Statistical analysis of variance of different pollens at a different level of plant taxa determination. The analyses at the Family and Genera level are made using ANOVA, the analyses at Species level are made using Wald statistics from LOG Generalized linear model.

		Microscopy			DNA Metabarcoding		
Taxonomic Level	Comparison Level	*F*	*df*	*p*	*F*	*df*	*p*
Family	Site	1.57	14; 39	0.13	2.66	14; 39	0.008
	Landscape category	2.05	2; 51	0.14	0.96	2; 51	0.39
Genera	Site	–	–	–	1.04	2; 51	0.36
	Landscape category						
		*Wald*. Stat.	*df*	*p*			
Species	Site	0.19	14; 427	0.65	–	–	–
	Landscape category	0.06	14; 427	0.80	–	–	–

## Data Availability

The databes was used for Bontšutšnaja, A.; Karise, R.; Mänd, M.; Smagghe, G. Bumble Bee Foraged Pollen Analyses in Spring Time in Southern Estonia Shows Abundant Food Sources. Insects 2021, 12, 922. https://doi.org/10.3390/insects12100922. The database contains the results of DNA metabarcoding (sequences, relative importances) and microscopy pollen count data.

## Supplementary Materials

The following are available online at https://www.mdpi.com/article/10.3390/insects12100922/s1, Figure S1: Examples of landscapes belonging to different categories. Red circles indicate 1 km radius around the bumble bee hives (center of the circles), Table S1: The strength of correlations based on *r*-values between land-use parameters and proportions of plant families, proportions of strawberry pollen and numbers of plant families in bumble bee corbicular pollen, Figure S2: Example of pollen grains (strawberry) seen under light microscopy, maginification x1000, Table S2: Kruskal-Wallis H test and relevant DF values of variation of proportions of plant families on both microscopy and DNA barcoding data at site and landscape level, Figure S3: The correlation matrixes of proportions of pollen grains counted with microscopy and relative abundance of reads of the results of DNA barcoding.
