# Supplementary material for: Bumble Bee Foraged Pollen Analyses in Spring Time in Southern Estonia Shows Abundant Food Sources
Source: Insects. 2021 Oct 9;12(10):922. doi: 10.3390/insects12100922 (PMC8538635; doi:10.3390/insects12100922)
Supplement: Supplementary file 1 [file insects-12-00922-s001.zip › Insects-1346492_Suppl_Figure_S1.pdf]

Insects-1346492

Figure S1. Examples of landscapes belonging to different categories. Red circles indicate 1 km radius around the bumble bee hives (centre of the circles)

Field

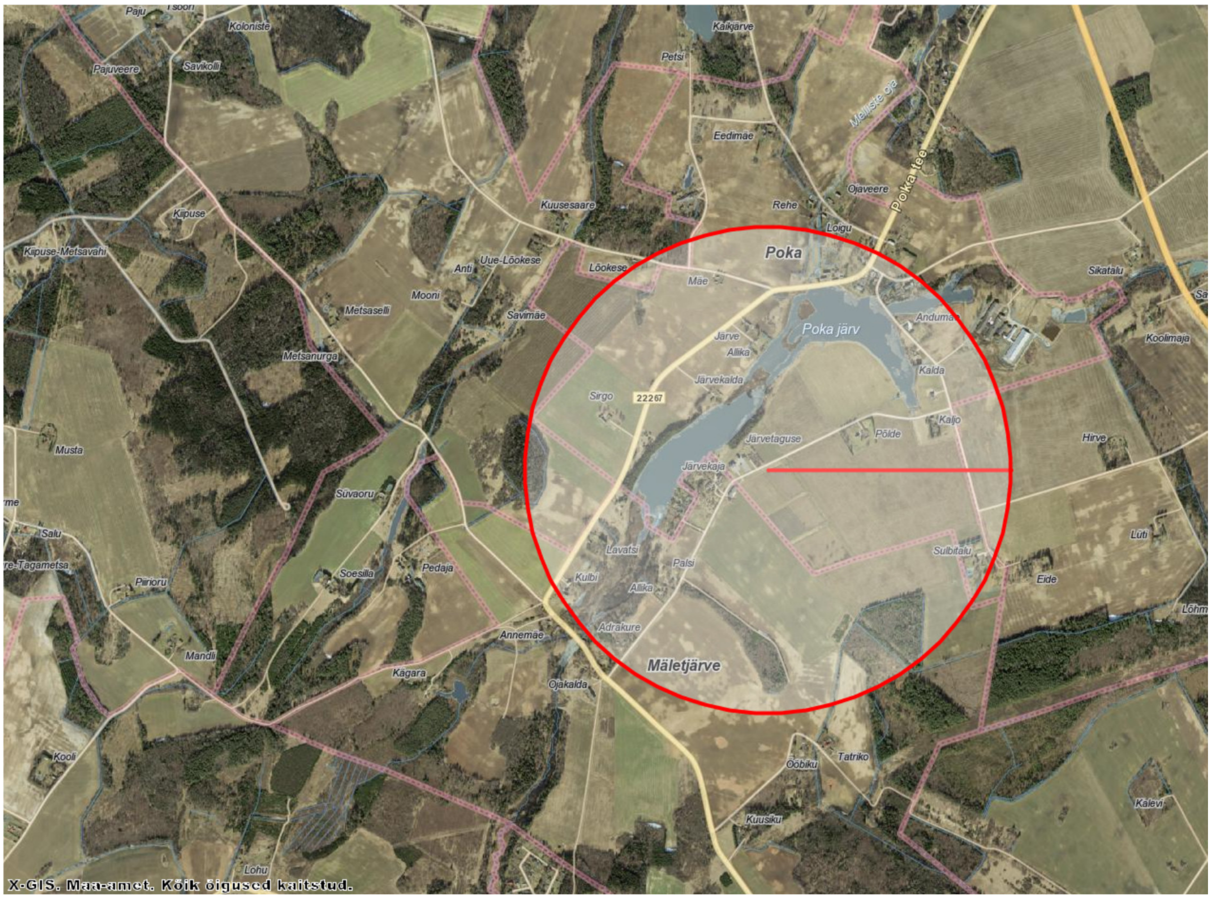

Forest

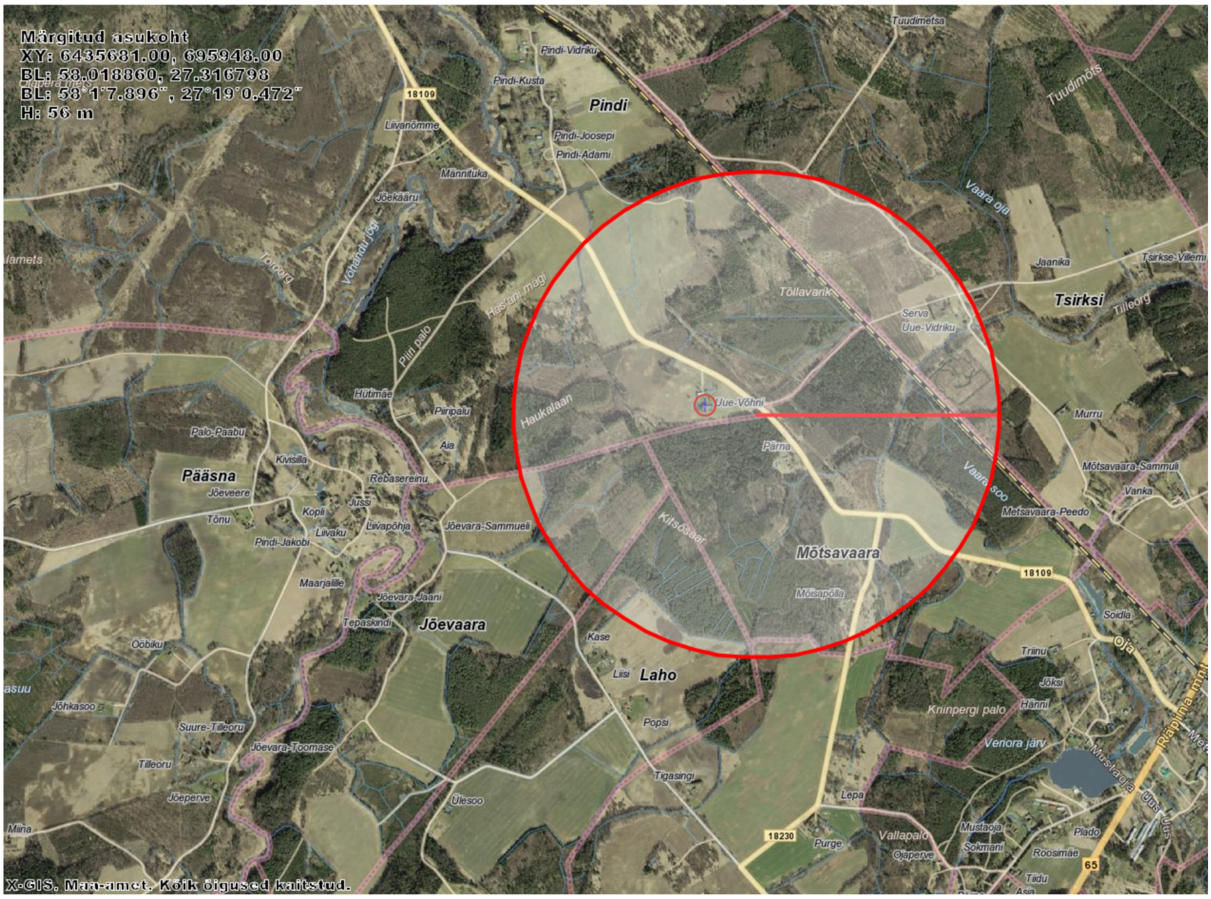
Märgitud asukoht
XY: 6435681.00, 695948.00
BL: 58.018860, 27.316798
BL: 58°1'7.896", 27°19'0.472"
H: 56 m
Pindi
Tsirksi
Päsna
Jõevaara
Laho
Mõtsavaara
Uue-Võhni
Tõllavank
Haukalaan
Pärna
Mõisapõllu
Kirsõsaar
Vaara soo
Vaara oja
18109
18230
65
X-GIS. Maa-amet. Kõik õigused kaitstud.

Orchard

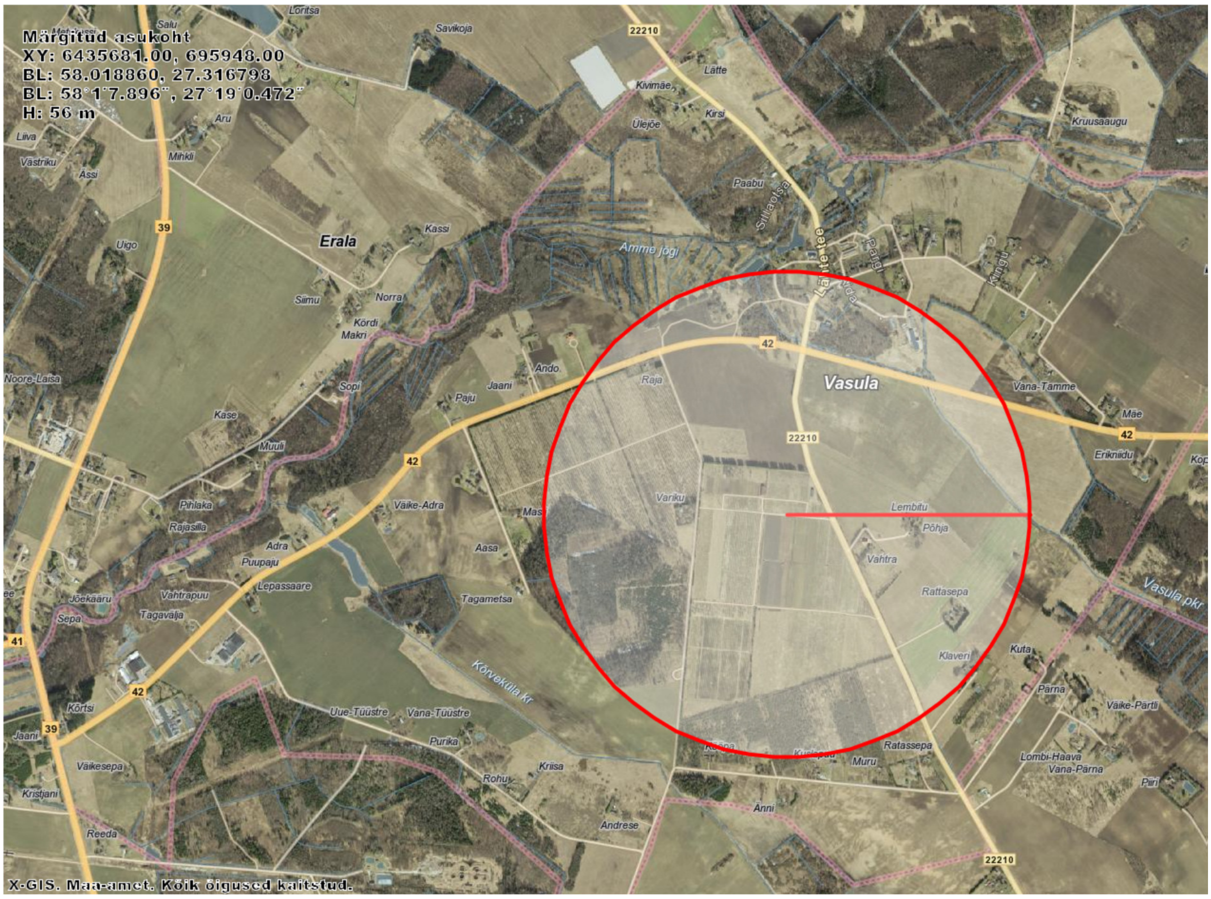

Märgitud asukoht
XY: 6435681.00, 695948.00
BL: 58.018860, 27.316798
BL: 58°1'7.896", 27°19'0.472"
H: 56 m
Erala
Vasula
Amme jõgi
Lätte tee
Vasula pkr
Kõrveküla kr
22210
42
39
41
Raja
Variku
Lembitu
Põhja
Vahtra
Rattasepa
Klaveri
Ratassepa
Muru
Anni
Andrese
Kuta
Parna
Vaike-Partli
Lombi-Haava
Vana-Parna
Vana-Tamme
Mäe
Erikniidu
Kruusaaugu
Lätte
Kivimäe
Ülejõe
Kirsi
Paabu
Savikoja
Kassi
Norra
Simu
Kördi
Makri
Sopi
Ando
Jaani
Paju
Muuli
Kase
Uigo
Mihkli
Assi
Aru
Salu
Lõritsa
Liiva
Västriku
Noora-Laisa
Pihlaka
Rajasilla
Adra
Puupaju
Lepassaare
Väike-Adra
Aasa
Tagametsa
Jõekaaru
Vahtrapuu
Sepa
Tagavälja
Kõrtsi
Jaani
Väikesepa
Kristiani
Reeda
Uue-Tüüstre
Vana-Tüüstre
Purika
Rohu
Kriisa
Piiri
X-GIS. Maa-amet. Kõik õigused kaitstud.
