# Supplementary material for: Bumble Bee Foraged Pollen Analyses in Spring Time in Southern Estonia Shows Abundant Food Sources
Source: Insects. 2021 Oct 9;12(10):922. doi: 10.3390/insects12100922 (PMC8538635; doi:10.3390/insects12100922)

Insects-1346492

Figure S3. The correlation matrixes of proportions of pollen grains counted with microscopy and relative abundance of reads of the results of DNA barcoding

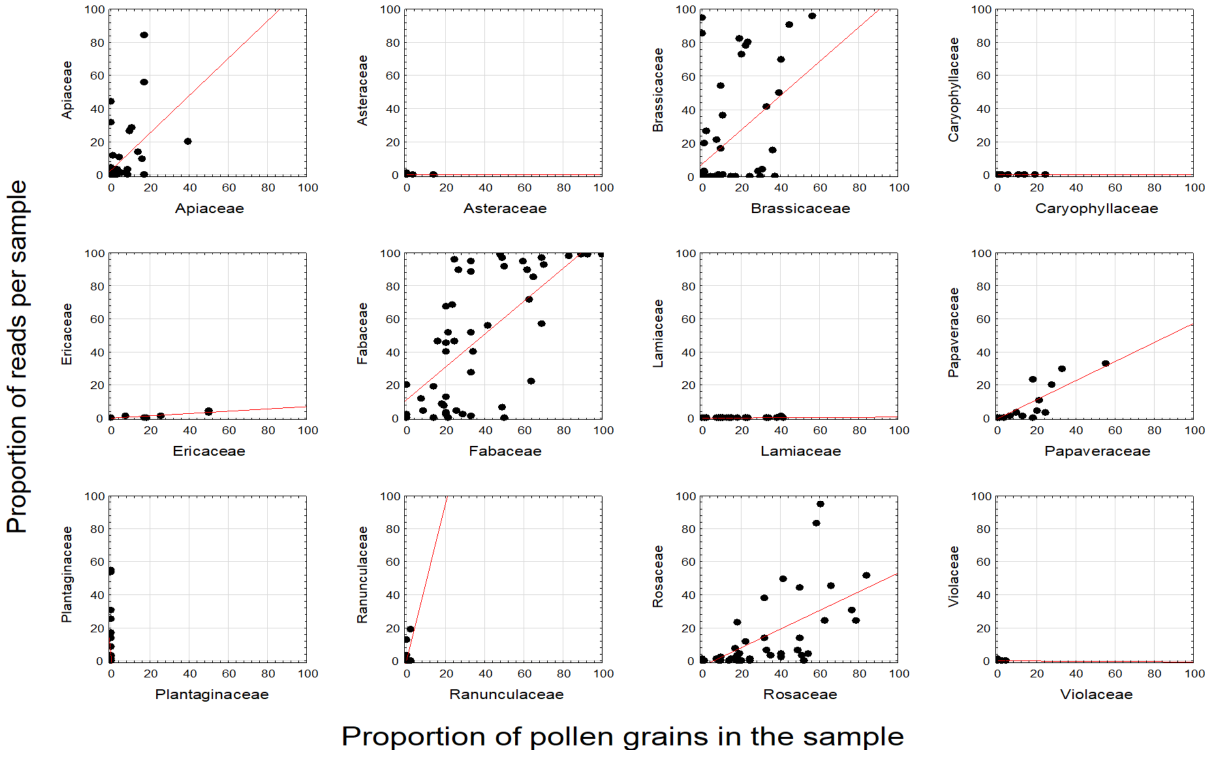

Supplement: Supplementary file 1 [file insects-12-00922-s001.zip › Insects-1346492_Suppl_Figure_S3.pdf]
