# Supplementary material for: Bumble Bee Foraged Pollen Analyses in Spring Time in Southern Estonia Shows Abundant Food Sources
Source: Insects. 2021 Oct 9;12(10):922. doi: 10.3390/insects12100922 (PMC8538635; doi:10.3390/insects12100922)
Supplement: Supplementary file 1 [file insects-12-00922-s001.zip › Insects-1346492_Suppl_Table_S2.pdf]

Table S2. Results of statistical analyses (Kruskal-Wallis H test and relevant DF values) of variation of proportions of plant families on both microscopy and DNA barcoding data at site and landscape level. P-values in bold indicate statistically significant differences.

	Microscopy				DNA metabarcoding			
	Site		Landscape category		Site		Landscape category	
**Plant familiy**	***H* (14;54)**	***P***	***H* (2;54)**	***P***	***H* (14;54)**	***P***	***H* (2;54)**	***P***
Apiaceae	27.63	**0.016**	7.08	**0.03**	29.21	**0.009**	5.53	0.06
Asteraceae	14.10	0.44	0.58	0.75	12.22	0.59	3.73	0.15
Brassicaceae	28.69	**0.012**	10.86	**0.004**	31.76	**0.004**	6.54	**0.04**
Caryophyllaceae	13.16	0.51	0.57	0.75	12.20	0.59	2.05	0.36
Ericaceae	40.10	**<0.001**	3.69	0.16	34.05	**0.002**	0.86	0.65
Fabaceae	32.67	**0.003**	3.96	0.14	36.70	**0.001**	7.07	**0.029**
Lamiaceae	22.17	0.075	5.13	0.08	28.04	**0.014**	2.61	0.27
Papaveraceae	32.68	**0.003**	5.27	0.07	31.62	**0.005**	10.93	**0.004**
Plantaginaceae	16.31	0.29	2.29	0.32	31.61	**0.005**	0.22	0.90
Ranunculaceae	6.98	0.93	0.20	0.91	14.87	0.38	4.23	0.12
Rosaceae	28.36	**0.013**	0.28	0.87	33.62	**0.002**	0.82	0.66
Violaceae	12.36	0.58	1.49	0.47	16.30	0.29	0.47	0.79
Boraginaceae					31.57	**0.005**	0.06	0.97
Orobanchaceae					38.43	**<0.001**	2.30	0.32
Primulaceae					38.81	**<0.001**	1.55	0.46
Salicaceae					19.98	0.13	**7.76**	**0.02**
