# Supplementary material for: Bumble Bee Foraged Pollen Analyses in Spring Time in Southern Estonia Shows Abundant Food Sources
Source: Insects. 2021 Oct 9;12(10):922. doi: 10.3390/insects12100922 (PMC8538635; doi:10.3390/insects12100922)
Supplement: Supplementary file 1 [file insects-12-00922-s001.zip › insects_1346492_Suppl_Table_S1.pdf]

insects-1346492

Table S1. The strength of correlations based on r-values between land use parameters and proportions of plant families, proportions of strawberry pollen and numbers of plant families in bumble bee corbicular pollen. The estimations are based on microscopy pollen grain counts and proportions of reads from DNA metabarcoding over pooled samples per hive, location and date. Red colours indicate negative and green colours positive correlations, the intensity of the colour increases towards values closer to -1 or 1. Pale colours indicate values close to zero. The statistically significant correlations are indicated with black frames.

		Microscopy					DNA barcoding				
		Woodland	Arable land	Grassland	Yard	Fruit gardens	Woodland	Arable land	Grassland	Yard	Fruit gardens
400 m radius	Apiaceae										
	Asteraceae										
	Brassicaceae										
	Caryophyllaceae										
	Ericaceae										
	Fabaceae										
	Lamiaceae										
	Papaveraceae										
	Plantaginaceae										
	Ranunculaceae										
	Rosaceae										
	Violaceae										
	Boraginaceae										
	Orobanchaceae										
	Primulaceae										
	Salicaceae										
	Fragaria x ananassa										
	No. of Families										
1000 m radius	Apiaceae										
	Asteraceae										
	Brassicaceae										
	Caryophyllaceae										
	Ericaceae										
	Fabaceae										
	Lamiaceae										
	Papaveraceae										
	Plantaginaceae										
	Ranunculaceae										
	Rosaceae										
	Violaceae										
	Boraginaceae										
	Orobanchaceae										
	Primulaceae										
	Salicaceae										
	Fragaria x ananassa										
	No. of Families										
	r-value	1	0,5	0	-0,5	-1					
